# Concentration–Response Analysis of the Combination of Pyronaridine and Piperaquine on Corrected QT Interval From a Randomized, Double‐Blind, Placebo‐Controlled Study in Healthy Adults of African Sub‐Saharan Origin

**DOI:** 10.1111/cts.70305

**Published:** 2025-07-21

**Authors:** Mathieu Felices, Isabelle Borghini‐Fuhrer, Nada Abla, Stephan Chalon

**Affiliations:** ^1^ PhinC Development Massy France; ^2^ MMV Medicines for Malaria Venture Geneva Switzerland

**Keywords:** chemoprevention, malaria, piperaquine, pyronaridine, QT interval, safety

## Abstract

A novel oral combination of the long‐acting antimalarials pyronaridine (PYR) and piperaquine (PQP) has potential for malaria chemoprevention. This single‐center randomized, double‐blind, placebo‐controlled study assessed the effects of PYR and PQP alone and when co‐administered on Fridericia‐corrected QT interval (QTcF). Between February 14, 2022, and May 31, 2022, thirty‐seven healthy black adults of African sub‐Saharan origin were enrolled and randomized to PYR + PQP (*n* = 15), PYR + placebo (*n* = 8), PQP + placebo (*n* = 8) or double placebo (*n* = 6) administered once daily (fasted) for 3 days at doses approved for malaria treatment. Triplicate digitalized electrocardiogram (ECG) recordings and pharmacokinetic samples were taken at matched timepoints. Concentration–response analysis was performed for QTcF changes from baseline (ΔQTcF), and the impact of PYR, PQP, and PYR + PQP administration on placebo‐corrected ΔQTcF (ΔΔQTcF) was assessed. The final qualified and validated concentration–QTc model included a linear component for PYR and an *E*_max_ component for PQP. The maximum predicted effect on ΔΔQTcF on day 3 was +4.94 msec (90% CI 0.338, 9.54) with PYR + placebo, +19.2 msec (14.6, 23.8) with PQP + placebo, and + 23.1 msec (18.5, 27.6) with PYR + PQP. As expected, PQP increased ΔΔQTcF above the regulatory threshold of concern (+10 msec), whereas PYR did not. The small additional increase in ΔΔQTcF with PYR + PQP coadministration was explained mainly by an increase in PQP *C*_max_ (1.4‐fold) versus monotherapy. In healthy adults, PYR + PQP coadministration does not appear to increase significantly the effect of PQP on ΔΔQTcF versus PQP administered alone. However, further studies are needed in malaria patients to confirm these findings in the target population.

**Trial Registration:** ClinicalTrials.gov identifier: NCT05160363; EudraCT number: 2021‐005698‐21

**Summary**

- What is the current knowledge on the topic
  ○ The antimalarials pyronaridine (PYR) and piperaquine (PQP) are being investigated as a potential new combination for malaria chemoprevention. PQP elicits a well‐documented exposure‐dependent prolongation of the QTc interval. PQP exposures are increased when administered with food, resulting in a larger QTc prolongation, and the drug should be dosed in the fasted state. When administered in the fasted state, PQP‐induced QTc interval prolongation has not been shown to be associated with an increased risk of arrhythmia or *torsade de pointes*. There are neither food restrictions on PYR administration nor clinically relevant QTc interval prolongation.
- What question did this study address?
  ○ What is the impact of PYR + PQP coadministration on Fridericia‐corrected QT interval (QTcF) compared to PYR or PQP alone? Data from a Phase I study of PYR + PQP coadministration was used to answer this question. A model describing the relationship between drug concentrations and QTcF changes from baseline in (ΔQTcF) was developed, validated, and used to calculate the impact of PYR, PQP, and PYR + PQP administration on placebo‐corrected ΔQTcF (ΔΔQTcF).
- What does this study add to our knowledge?
  ○ Relative to PQP alone, PYR + PQP coadministration resulted in a small increase in ΔΔQTcF, explained mainly by an increase in PQP *C*_max_. The limited contribution of PYR and the saturable PQP effect after the initial dose suggest that the cardiac risk of PYR + PQP is similar to that of PQP administered alone in a fasted state.
- How might this change clinical pharmacology or translational science?
  ○ The study illustrates that it is possible to design Phase 1 studies that allow efficient evaluation of drug combination effects on the QT interval, consistent with regulatory standards. Concentration–QTc modeling was used to assess a novel investigational combination of two approved antimalarial drugs at therapeutic doses and allowed quantification of the effects on the QT interval for a potential chemopreventive dosing regimen. This study provides a template for concentration–QTc modeling of antimalarial drug combinations.

## 1 Introduction

There were an estimated 263 million malaria cases globally in 2023, with children and pregnant women most at risk of adverse outcomes [1, 2]. Chemoprevention uses the full therapeutic course of antimalarial medicines at prespecified times, irrespective of infection status, to treat existing sub‐clinical infection and prevent new infection in endemic regions [1]. Sulfadoxine‐pyrimethamine (SP) is recommended for the intermittent preventive treatment of malaria in pregnancy, whereas chemoprevention in children uses SP + amodiaquine (SPAQ) [1]. Chemoprevention is currently effective in most African regions, but is threatened by the spread of high‐level resistance to SP and AQ [2, 3]. Thus, new chemopreventive medicines are needed to protect at‐risk populations [4].

Recombination of existing antimalarial drugs facilitates the rapid development of new chemopreventive therapies [4]. Pyronaridine (PYR) and piperaquine (PQP—formulated for oral administration as the phosphate salt) are components of fixed‐dose combinations with artesunate (PYR‐AS; Pyramax) and dihydroartemisinin (DHA‐PQP; Eurartesim), approved for the treatment of uncomplicated malaria [1]. A combination of PYR + PQP may have potential for malaria chemoprevention provided an acceptable risk:benefit ratio can be demonstrated. Both drugs are dosed once daily for 3 days, have different mechanisms of action [5, 6], and half‐lives are between 14 to 18 days for PYR and 24 days for PQP [5, 7], which is compatible with monthly dosing. Neither drug has teratogenic liabilities [8, 9], suggesting possible utility in women of childbearing potential and during pregnancy [10, 11]. There are no concerning overlaps in the known adverse event profiles that preclude combination [8, 9].

Previous studies report that PQP causes a linear exposure‐dependent prolongation of the Fridericia‐corrected QT interval (QTcF) in healthy volunteers and malaria patients [12, 13, 14]. Food increases PQP exposure, and consequently, QTcF prolongation, so the drug must be administered in the fasted state [5, 12]. When administered correctly with regard to food and other potential risk factors for QTcF prolongation, PQP was not associated with an increased risk for ventricular tachyarrhythmias or *torsade de pointe* [12, 13, 15, 16]. Contrastingly, PYR can be taken with or without food, with no clinically relevant QTcF prolongation at therapeutic doses for acute uncomplicated malaria [9, 17].

A Phase I study for the PYR + PQP combination was conducted in healthy black adults of African sub‐Saharan origin [18]. With the dosing regimens registered for the treatment of uncomplicated malaria, there were no clinically concerning safety signals following PYR + PQP coadministration compared with the individual drugs plus placebo [18]. In a standard categorical analysis, mild QTcF prolongation (> 450 to < 480 msec or > 30 to < 60 msec change versus baseline) was reported for 4/23 participants receiving PQP‐containing regimens [18], consistent with previous reports [12]. PYR + PQP coadministration increased PYR maximum blood concentrations (*C*_max_) on day 3 by 1.3‐fold and PQP *C*_max_ in plasma by 1.4‐fold [18].

Since 2015, regulators have allowed concentration–QTc modeling—usually performed in healthy volunteers enrolled in early drug development programs—as an alternative to thorough QT studies, which are typically conducted in parallel to Phase 2/3 trials [19]. We report the development of a concentration–QTc model for PYR, PQP, and PYR + PQP using pharmacokinetic (PK) and electrocardiogram (ECG) data from the Phase I study [18]. It was expected that PQP administered in a fasting state over 3 days would induce a clinically relevant QTc prolongation (> 10 msec) with a maximum effect achieved on day 3 [12], while no or limited effects of PYR on QTc were anticipated [17]. The effect of PYR + PQP on QTc has not been previously investigated.

## 2 Methods

### 2.1 Study Drugs

Study drugs were PYR 180 mg tablets and matched placebo (Shin Poong Pharm. Co. Ltd., Ansan, Korea) and PQP 320 mg tablets and matched placebo (Piramal Pharma Ltd., Mumbai, India). PYR and PQP dosing regimens were as for the approved antimalarial drugs PYR‐AS and DHA‐PQP, that is, once daily for 3 days by body weight: PYR 540 mg (50–< 65 kg) and 720 mg (≥ 65 kg); PQP 960 mg (50–< 75 kg) and 1280 mg (≥ 75 kg).

### 2.2 Study Design

The objective of the current concentration–effect analysis was to use a model‐based approach to determine the relationship between PYR or PQP drug concentrations and QTc interval following administration of PYR‐placebo, PQP‐placebo, or PYR + PQP. Data were derived from a Phase I, single‐center, randomized, double‐blind, placebo‐controlled, parallel group study. Thirty‐seven participants were enrolled and randomized to PYR + PQP (*n* = 15), PYR + placebo (*n* = 8), PQP + placebo (*n* = 8) or placebo + placebo (*n* = 6), stratified to include at least two participants at each dosing level. The study was conducted between 14th February 2022 and 31st May 2022 at Richmond Pharmacology Ltd., London, UK. Clinical and PK findings have been reported [18].

### 2.3 Participants

Healthy males and females (not pregnant or breastfeeding) of self‐reported black ethnicity and sub‐Saharan ancestry were eligible if they were aged between 18 and 45 years, with bodyweight ≥ 50 kg and body mass index 18–28 kg/m^2^. Participants were excluded if they had any clinically significant ECG or Holter abnormalities in rhythm, conduction, or morphology, or any clinically important abnormalities that could interfere with QTc interval interpretation, including sinus node dysfunction, clinically significant PR interval prolongation (> 220 msecs), second‐ or third‐degree atrioventricular block, sustained cardiac arrhythmias, symptomatic arrhythmia (except isolated extra‐systoles) or abnormal T‐wave morphology or QTcF > 450 msec. Other key exclusion criteria were any clinically relevant illness or surgery within 4 weeks prior to dosing, any abnormal vital signs, physical examination, or safety laboratory values, or any clinically relevant medical history. Full exclusion criteria have been published [18].

### 2.4 Procedures and Assessments

Participants received their allocated treatments on days 1, 2, and 3 following a ≥ 3 h fast and were required to fast for ≥ 4 h after dosing. PK samples were obtained on day 1 pre‐dose, then post‐dose at 1, 2, 3, 4, 5, 6, 7, 8, and 12 h; on day 2 pre‐dose, then post‐dose at 4, 6, and 12 h; and on day 3 pre‐dose, then post‐dose at 1, 2, 3, 4, 5, 6, 7, 8, 12, 24 (day 4), 72 (day 6), 120 (day 8), 288 (day 15) and 456 h (day 22). Analyte concentrations were determined using validated methods with a limit of quantification of 1.00 ng/mL in whole blood for PYR and 1.00 ng/mL in plasma for PQP by Swiss Bioquant (Reinach, Switzerland) [18].

Triplicate ECGs were measured after a rest period of ≥ 10 min in the supine position at times matched to the blood/plasma sample times, as well as at screening and on day −1 and then on days 1 and 2 and 3 at −1.5, −1 and −0.5 h pre‐dose, and additionally on day 3 at −5 min pre‐dose, 48 h (day 5) and on day 30. As part of the sentinel dosing strategy, two initial study participants in each cohort had triplicate ECGs at 96 h following the day 3 dose before progressing to dose the other participants in the cohort.

### 2.5 Analysis Dataset

The analysis population included all participants who received at least one dose of study medication and who had at least one valid pre‐dose ECG assessment, one valid post‐dose ECG assessment, and one measurable PQP or PYR concentration matching the post‐dose ECG assessments, or who received a placebo. For each study participant, PQP and PYR concentrations matching the ECG records were included in the analysis. Participants receiving a placebo had concentration values set to 0. There was no imputation for missing ECG or concentration data.

Baseline ECG quantitative measurements were defined as the average of values of triplicate ECGs performed at 1.5, −1, and −0.5 h pre‐dose on day 1. Post‐dose ECG variables were the mean of the triplicate values at each time point for each subject. QT interval was corrected for heart (HR) using Fridericia's method [20] if the absolute mean time‐matched difference to placebo was < 10 bpm for all timepoints for PYR, PQR, and PYR + PQP; otherwise, Bazett's formula [21] was to be used.

### 2.6 Outcomes

For PQP and PYR concentrations, the principal endpoint was changes from baseline in QTcF (ΔQTcF) that were adjusted for placebo, leading to a ‘double correction’ (ΔΔQTcF) and associated 90% confidence intervals (CI), computed from a concentration–QTc model. The impact of the drug on QTc prolongation was considered below the threshold of regulatory concern if the upper bound of the 90% CI of ΔΔQTcF predicted at the highest *C*_max_ was < 10 msec.

### 2.7 Concentration–QTc Model

Model development and analysis were consistent with recommendations from a scientific white paper on concentration–QTc modeling and the International Council for Harmonization guidelines [19, 22, 23]. Statistical analysis was performed using SAS software version 9.4.

A mixed linear model was assumed as a starting point, with fixed‐effect parameters of intercept, linear components (slope) for PQP and PYR concentrations, influence of baseline (centered on mean), a treatment‐specific intercept, and day‐by‐time effect on intercept, as previously published (Equation S1) [22]. Subject‐specific random effects (between‐subject variability) were added on intercept and slope parameters with an unstructured covariance matrix [22].

Model assumptions were assessed using graphical exploration of the dataset, that is no drug effect on HR, QTc interval independent of HR, direct drug effect on ΔQTc without hysteresis, and a linear effect of drug concentrations on QTc [22]. The detection and impact of hysteresis were further evaluated using the exposure‐normalized Glomb‐Ring Index (enGRI), with an enGRI > 2 considered impactful [24]. If the linear relationship between ΔQTcF and any analyte could not be accepted, the linear contribution could be replaced with an *E*_max_ contribution, or alternative models could be investigated [25].

Final model selection was based on Akaike information criterion (AIC). The final model was evaluated using goodness‐of‐fit plots and visual predictive checks comparing observed ΔQTc with predicted ΔQTc (median, 5th and 95th percentile with simulated 90% prediction interval), and model‐predicted ΔQTc with 90% CI compared to mean ΔQTc with 90% CI by deciles of concentrations (quantile plot). Model parameter estimates with standard error (SE) and 2‐sided 95% CI were derived, and their statistical significance assessed by student *t*‐test (*p* = 0.05 significance boundary). Random effects not supported by the model or resulting in null estimates were removed to avoid non‐convergence problems. A significant treatment effect suggested model mis‐specification (e.g., nonlinearity or hysteresis).

The final model was used to derive the ΔΔQTcF, SE, and 2‐sided 90% CIs for the geometric mean (GM) *C*_max_ for PYR, PQP, and PYR + PQP at days 1 and 3. If PYR and PQP were included in the final model, the *C*_max_ and *T*_max_ of both were determined, and the ΔΔQTcF and 90% CIs for the combination were computed for each analyte at their *C*_max_, accounting for the contribution of the other analyte at the GM *C*_max_ computed at their *T*_max_. The final model was used to conduct an exploratory analysis simulating a chemoprevention dosing regimen of PYR + PQP administered once daily for 2 days on the first day of each month for 6 months.

### 2.8 Ethics Statement

The study conformed to the Declaration of Helsinki, Good Clinical Practice, and all applicable laws. Informed written consent was obtained from all participants. Ethical approval was obtained from South Central–Berkshire B Ethics Committee, and the protocol was approved by the United Kingdom Medicines and Healthcare Products Regulatory Agency. The study protocol has been published [18].

## 3 Results

### 3.1 Data

All 37 enrolled participants were included in the analysis set (Table 1). Baseline data were consistent across the treatment groups (previously published) [18]. Doses ranged between 8.1–11.0 mg/kg for PYR and 13.1–18.9 mg/kg for PQP (Table 1). Concentration−time plots and PK data are published [18].

**TABLE 1:** Summary of the dataset used for the concentration–QTc analysis.

Treatment	Participants	PYR dose (mg/kg)	PQP dose (mg/kg)	ECG	PYR samples	PQP samples	PYR − ECG matched pairs	PQP − ECG matched pairs
Total	37	9.4 (0.9) [8.1–11.0]	15.4 (1.7) [13.1–18.9]	1329	1069	1070	1032	1033
PYR + PQP	15	9.5 (0.8) [8.1–10.7]	15.2 (1.7) [13.1–18.9]	540	434	435	419	420
PYR + placebo	8	9.4 (1.1) [8.1–11.0]	—	286	230	230	222	222
PQP + placebo	8	—	15.7 (1.7) [13.6–18.9]	287	232	232	224	224
Placebo+placebo	6	—	—	216	173	173	167	167

*Note:* Data are number, except for dose range which is mean (standard deviation) [range].

### 3.2 Graphical Exploratory Analysis

The HR, ΔHR, and ΔΔHR time course showed variations across time, but similar trends between treatments and no difference from placebo (Figure S1), suggesting that the four treatment groups exhibited very similar circadian variations for HR, with no trends in ΔHR related to PQP or PYR exposure that could affect QT correction. There was no evidence of a dependency between HR and QTcF, confirming the appropriateness of Fridericia's correction (Figure S2). There was no hysteresis for PYR or PQP administered with placebo (enGRI −1.05 to 0.54), but with PYR + PQP, counter‐clockwise loops supported by enGRI > 2 were observed for PQP and PYR (Figure S3). However, as there was no evidence of hysteresis with the treatments administered alone, these effects were considered as likely due to the contribution of PQP on PYR evaluation (and vice versa), rather than a delayed effect.

The time course for QTcF, ΔQTcF, and ΔΔQTcF showed increases in QTcF after each administration (Figure S4), with mild increases observed for PYR administered alone (< 10 msec) and increases of greater amplitude (> 10 msec) for PQP alone and PQP + PYR compared with placebo. The effect of PYR + PQP on ΔQTcF was probably mostly driven by PQP, though both PQP and PYR concentrations were increased following coadministration versus each drug singly. A linear rising phase of ΔQTcF was observed for PQP alone or in combination with PYR, up to about 500 ng/mL; thereafter, the relationships appeared to flatten and move away from linearity to reach different plateaus for the two treatments (Figure S5).

### 3.3 Model Development

Starting from the linear model (Equation S1), a review of the diagnostic and validation plots indicated a bias at the highest PQP concentration and a curvature not accounted for in the model (Figure S6), confirming that the PQP contribution to ΔQTcF was unlikely to be linear. The model was refined to include a linear contribution for PYR and a non‐linear *E*_max_ contribution for PQP. Fixed effects were intercept, specific treatment shift for each treatment investigated, baseline covariate, slope for PYR concentrations, *E*_max_, and EC_50_ for PQP concentrations, with the day‐by‐time interaction maintained (Equation S2). The final model provided the lowest AIC (6939.1).

**FIGURE 1:**
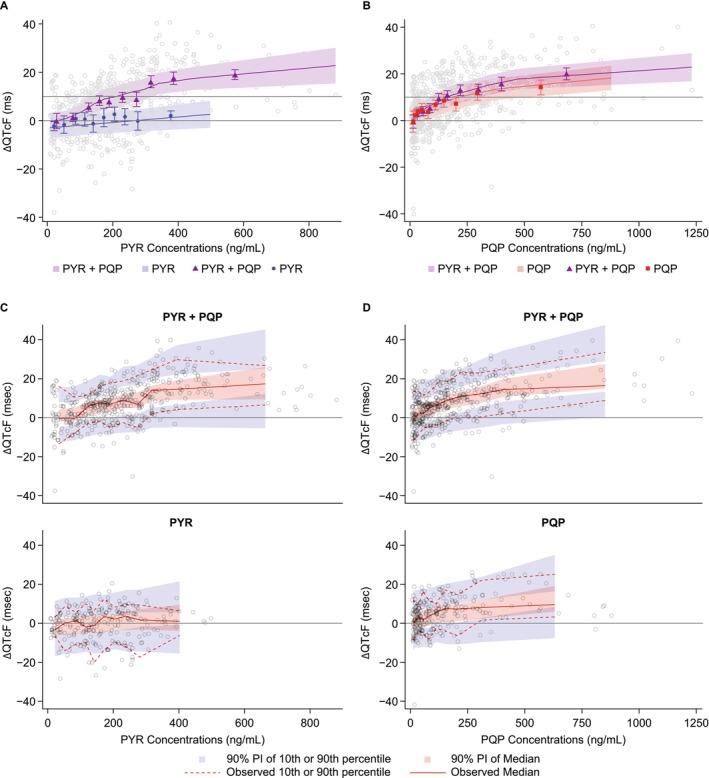
Final concentration–ΔQTcF model validation by mean estimated and observed ΔQTcF (90% CI) by deciles of concentrations for (A) pyronaridine (PYR) and (B) piperaquine (PQP); and by visual predictive checks by deciles of concentrations for (C) PYR and (D) PQP. Treatments: PYR + placebo; PQP + placebo; PYR + PQP; placebo + placebo, once daily for 3 days, dosed by bodyweight: PYR 540 mg (50–< 65 kg) or 720 mg (≥ 65 kg); PQP 960 mg (50–< 75 kg) or 1280 mg (≥ 75 kg).

Estimated key model parameters are summarized in Table 2. Overall, the global intercept was estimated at −3.24 (95% CI −7.82, 1.35) msec and not statistically significant. Treatment‐specific shifts were estimated at 1.93 (−3.38, 7.24), 3.50 (−2.04, 9.03), and 2.55 (−2.71, 7.81) for PYR, PQP, and PYR + PQP treatments, respectively. None were statistically significant, counterbalancing the negative contribution of the placebo participants. The estimated slope for PYR contribution was 0.0094 (0.0010, 0.0177) msec per ng/mL, indicating a weak but statistically significant linear increase of QTcF in relation to PYR concentrations. The model parameters for PQP contribution were 25.7 (17.3, 34.0) msec for *E*_max_ and 300 ng/mL (58.2, 542) for EC_50_; both statistically significant. The baseline covariate and some of the day‐by‐time components were significant, but these terms were included in the model to capture the variability, are not relevant for the interpretation of the model and will not be discussed further (Table S1). Among the between‐subject random effects, only that for the intercept was supported by the data. The residual variability was weak (SD = 6.5 msec). The diagnostic and goodness‐of‐fit plots did not show any trend suggesting model mis‐specification (Figure S7). Visual predictive checks were consistent with a correctly specified model, and the negative bias and curvature observed for PQP concentrations with the linear model (Figure S6) were not evident in the final model (Figure 1).

**TABLE 2:** Summary of final concentration–QTcF model parameter estimates.

Parameters	Estimate (SE)	95% CI	*p*
$\theta_{0}$ Intercept: placebo, Day 3, Time 456 h (msec)	−3.24 (2.26)	−7.82, 1.35	0.1603
$\theta_{3,\mathrm{PYR}}$ Treatment‐specific intercept PYR (msec)	1.93 (2.61)	−3.38, 7.24	0.4661
$\theta_{3,\mathrm{PQP}}$ Treatment‐specific intercept PQP (msec)	3.50 (2.72)	−2.04, 9.03	0.2079
$\theta_{3,\mathrm{PYR}+\mathrm{PQP}}$ Treatment‐specific intercept PYR + PQP (msec)	2.55 (2.59)	−2.71, 7.81	0.3308
$\theta_{6}$ Baseline covariate (msec)	−0.116 (0.046)	−0.209, −0.024	0.0154
$\theta_{2}$ PYR slope (msec per ng/mL)	0.0094 (0.0041)	0.0010, 0.0177	0.0287
$\theta_{1}^{1}$ PQP *E*_max_ (msec)	25.7 (4.10)	17.3, 34.0	< 0.0001
$\theta_{2}^{1}$ PQP EC_50_ (ng/mL)	300 (119)	58.2, 542	0.0165
**Variance components**			
Between‐subject variance (intercept)	19.3 (5.55)	7.98, 30.5	0.0014
Between‐subject variance (slope PYR)	0.0001 (0.0001)	−0.0001, 0.0004	0.1993
Covariance (intercept, *E*_max_ PQP)	0.117 (6.74)	−13.6, 13.8	0.9863
Covariance (slope PYR, *E*_max_ PQP)	−0.027 (0.032)	−0.092, 0.038	0.4020
Between‐subject variance (*E*_max_ PQP)	10.4 (12.9)	−15.9, 36.7	0.4259
Residual	42.1 (2.09)	37.9, 46.4	< 0.0001

*Note:* For full information on final model parameter estimates, see Table S1.

Abbreviations: CI, confidence interval; SE, standard error.

### 3.4 Evaluation of Concentration Effect on ΔΔQTcF

**FIGURE 2:**
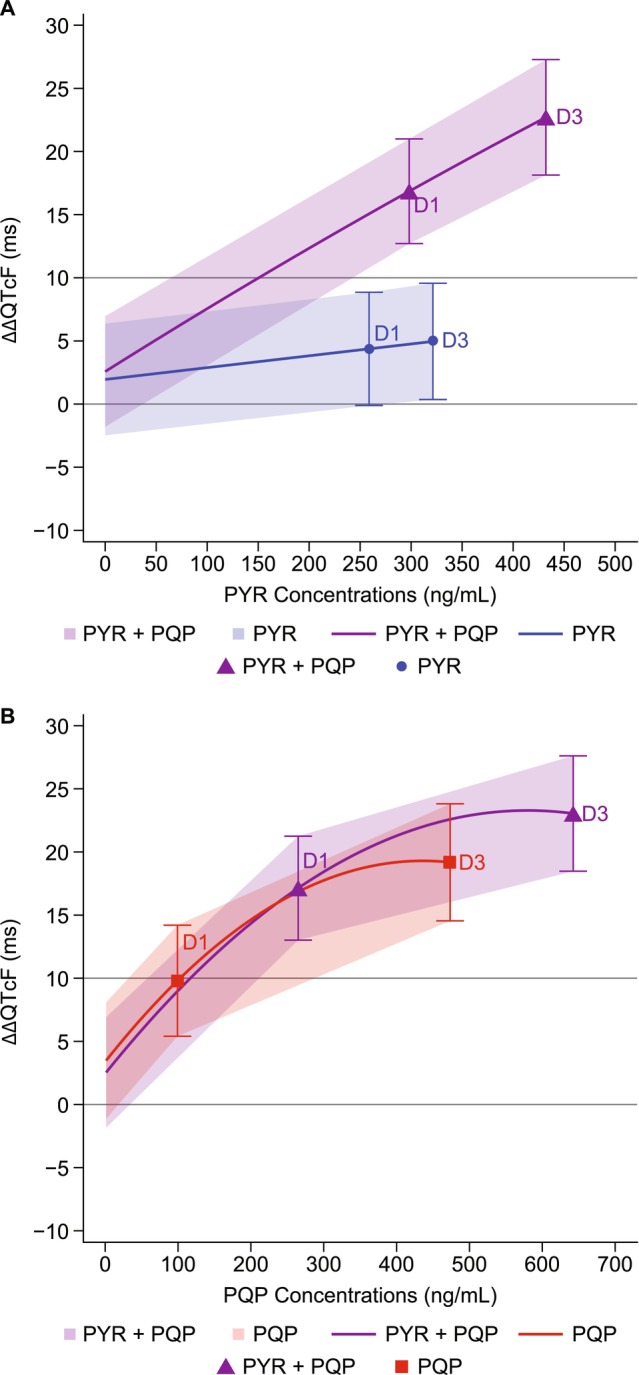
Model‐predicted ΔΔQTcF (90% CI) from the final concentration–ΔQTcF model, which included both PYR and PQP concentrations as covariates, across different treatment arms and exposure levels, at geometric mean *C*_max_ for (A) pyronaridine (PYR) and (B) piperaquine (PQP). Each plot shows predicted ΔΔQTcF versus the concentration of the indexed drug (x‐axis), with the co‐administered drug held at its Day 3 geometric mean *C*_max_. For example, panel A shows predictions at the geometric mean PYR *C*_max_ on Day 3, with piperaquine (PQP) concentration fixed at the mean PQP concentration observed at the time of PYR *C*_max_. Curves and shaded bands represent model‐based predictions for each drug individually and in combination (PYR + PQP). Data are shown for Day 1 and Day 3 (D1, D3) for each treatment condition. Treatments: PYR + placebo; PQP + placebo; PYR + PQP; placebo + placebo, once daily for three days, dosed by body weight: PYR 540 mg (50–< 65 kg) or 720 mg (≥ 65 kg); PQP 960 mg (50–< 75 kg) or 1280 mg (≥ 75 kg).

The final model parameters were used to compute predicted ΔΔQTcF (SE) and 90% CIs at the GM *C*_max_ on day 1 and day 3 (Methods S1). The predicted ΔΔQTcF and 90% CIs are presented in Table 3 and shown graphically by compound in Figure 2.

**TABLE 3:** Final concentration–QTcF model prediction of effects of pyronaridine (PYR) and piperaquine (PQP) concentrations on ΔΔQTcF for each treatment and day.

Treatment	Day	Analyte	GM *C*_max_, ng/mL	Estimated ΔΔQTcF (SE), msec	90% CI
PYR + placebo	1	PYR	258.7	4.35 (2.65)	−0.128, 8.83
PYR + placebo	3	PYR	321.4	4.94 (2.72)	0.338, 9.54
PYR + PQP	1	PYR	298.1	16.8 (2.45)	12.7, 21.0
PYR + PQP	3	PYR	432.1	22.7 (2.70)	18.1, 27.2
PQP + placebo	1	PQP	98.1	9.82 (2.60)	5.42, 14.2
PQP + placebo	3	PQP	473.3	19.2 (2.74)	14.6, 23.8
PYR + PQP	1	PQP	264.2	17.2 (2.44)	13.0, 21.3
PYR + PQP	3	PQP	642.6	23.1 (2.70)	18.5, 27.6

Abbreviations: *C*_max_, maximum blood concentration for PYR and plasma concentration for PQP; GM, geometric mean; SE, standard error; ΔΔQTcF, placebo‐corrected change from baseline in QTcF.

For PYR + placebo, despite a mild increase between day 1 and day 3 related to PYR concentrations, the ΔΔQTcF upper bound of the 90% CI remained < 10 msec on day 1 (4.35 msec [−0.128; 8.83]) and day 3 (4.94 msec [0.338; 9.54]) (Figure 2A). With PYR in combination with PQP, the predicted effects on ΔΔQTcF were > 10 msec on day 1 (16.8 msec [12.7; 21.0]) and day 3 (22.7 msec [18.1; 27.2]), driven primarily by the contribution of PQP. Although there was an increase in PYR *C*_max_ following coadministration, the impact on ΔΔQTcF was limited, consistent with the linear model for PYR.

For PQP + placebo, the ΔΔQTcF upper bound of the 90% CI was > 10 msec on day 1 (9.82 msec [5.42; 14.2]) and day 3 (19.2 msec [14.6; 23.8]), and the non‐linear pattern of the concentration–response relationship was evident (Figure 2B). With PQP in combination with PYR, the predicted effect was increased versus PQP + placebo on both day 1 (17.2 [13.0, 21.3]) and day 3 (23.1 [18.5, 27.6]). This increase in ΔΔQTcF was mostly explained by the higher PQP *C*_max_ when administered in combination with PYR, versus PQP + placebo on both day 1 (264.2 ng/mL vs. 98.1 ng/mL) and day 3 (642.6 ng/mL vs. 473.3 ng/mL). However, the effect plateaued at higher concentrations, limiting the increase in ΔΔQTcF (Figure 2B).

### 3.5 Evaluation of Potential Chemopreventive Regimen

**FIGURE 3:**
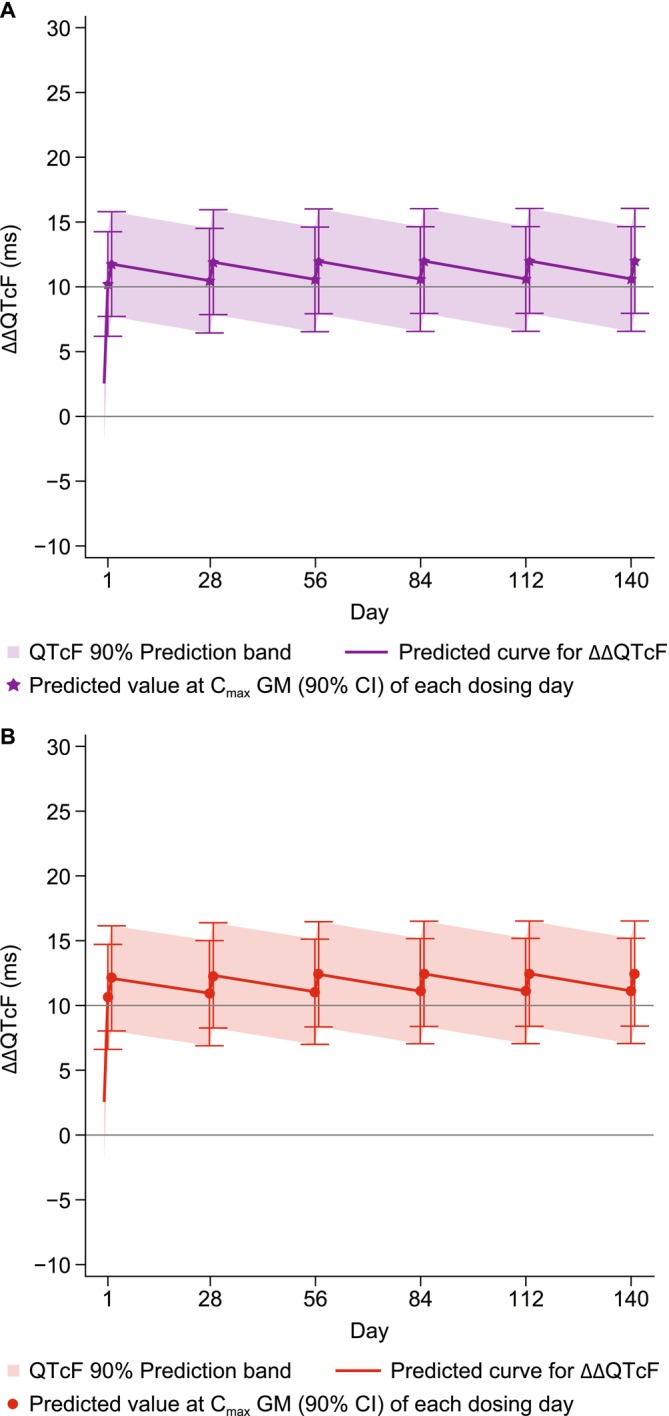
Predicted ΔΔQTcF at the simulated *C*_max_ following coadministration of pyronaridine (PYR) + piperaquine (PQP) once daily for 2 days on the first day of each month for 6 months for (A) PYR and (B) PQP, dosed by bodyweight: PYR 540 mg (50–< 65 kg) or 720 mg (≥ 65 kg); PQP 960 mg (50–< 75 kg) or 1280 mg (≥ 75 kg). Each panel displays the drug‐specific predicted QTcF effect based on separate concentration–ΔQTcF models for PYR and PQP. Data are presented as predicted values at geometric mean *C*_max_ (points with 90% CI) and predicted curves with 90% prediction intervals.

The final model was used to conduct an exploratory analysis of the effect of PYR‐PQP on ΔΔQTcF when administered once daily on the first 2 days of each month for 6 months (Figure 3). There was a slight increase in the predicted ΔΔQTcF at the final dose versus the initial dose, reaching a maximum of 12.5 msec (90% CI 8.4, 16.5) at the PQP *C*_max_ (Table 4).

**TABLE 4:** Predicted ΔΔQTcF at the simulated exposure for PYR‐PQP dosed once daily for 2 days each month for six consecutive months, dosed by bodyweight: PYR 540 mg (50–< 65 kg) or 720 mg (≥ 65 kg); PQP 960 mg (50–< 75 kg) or 1280 mg (≥ 75 kg).

Month	Day	ΔΔQTcF predicted at PQP *C*_max_		ΔΔQTcF predicted at PYR *C*_max_	
		Estimate (SE)	90% CI	Estimate (SE)	90% CI
1	1	10.7 (2.40)	(6.60, 14.7)	10.2 (2.39)	(6.19, 14.3)
1	2	12.1 (2.40)	(8.03, 16.2)	11.8 (2.39)	(7.72, 15.8)
2	28	10.9 (2.40)	(6.89, 15.0)	10.5 (2.39)	(6.45, 14.5)
2	29	12.3 (2.40)	(8.26, 16.4)	11.9 (2.39)	(7.87, 16.0)
3	56	11.1 (2.40)	(6.99, 15.1)	10.6 (2.39)	(6.54, 14.6)
3	57	12.4 (2.40)	(8.35, 16.5)	12.0 (2.39)	(7.93, 16.0)
4	84	11.1 (2.40)	(7.04, 15.2)	10.6 (2.39)	(6.56, 14.6)
4	85	12.4 (2.40)	(8.38, 16.5)	12.0 (2.39)	(7.95, 16.0)
5	112	11.1 (2.40)	(7.06, 15.2)	10.6 (2.39)	(6.57, 14.7)
5	113	12.5 (2.40)	(8.40, 16.5)	12.0 (2.39)	(7.96, 16.1)
6	140	11.1 (2.40)	(7.06, 15.2)	10.6 (2.39)	(6.58, 14.7)
6	141	12.5 (2.40)	(8.40, 16.5)	12.0 (2.39)	(7.96, 16.1)

Abbreviation: SE, standard error.

## 4 Discussion

This Phase 1 study evaluated the effects of PYR, PQP, and PYR + PQP on the QT interval in healthy adults using a concentration–ΔQTcF model. The final model enabled computation of ΔΔQTcF for each treatment individually and in combination, as per ICH E14 guidance [19, 23]. This study informs risk:benefit assessment and illustrates a robust approach for evaluating QT interval prolongation risk early in the clinical development of antimalarial combinations.

As expected, PYR administered alone (8.1–11.0 mg/kg, fasted) showed no clinically relevant QTcF prolongation, with the upper bound of the 90% CI for ΔΔQTcF remaining below the regulatory threshold (10 msec) on days 1 and 3. This aligns with previous observations in malaria patients [13].

As anticipated, with PQP alone (13.1–18.9 mg/kg, fasted) ΔΔQTcF exceeded the threshold on day 1 and day 3 (19.2 msec [90% CI: 14.6, 23.8]), consistent with prior reports in healthy adults (21.0 msec [15.7, 26.4]) [12]. The observed PQP *C*_max_ (473.3 ng/mL) was similar to previously reported values under fasted conditions (461 ng/mL with DHA‐PQP) [12].

Coadministration of PYR (8.1–11.0 mg/kg) and PQP (13.1–18.9 mg/kg) led to a mild increase in day 3 ΔΔQTcF to 23.1 msec (90% CI 18.5, 27.6). However, this remained within the upper bound of the 90% CI for ΔΔQTcF observed with PQP + placebo, and the QTcF effect of the combination was comparable to PQP alone. This occurred despite a 1.4‐fold increase in day 3 PQP *C*_max_ and a 1.3‐fold increase in PYR *C*_max_, due to a flattening of the concentration−ΔQTcF relationship at higher PQP concentrations, with minimal additional effect from PYR.

The findings suggest that PYR + PQP co‐administered once daily for 3 days had no significant impact on QT prolongation risk relative to PQP + placebo in healthy adults. Supporting these predictions, categorical QTcF analysis revealed no values exceeding 480 msec, no baseline changes > 60 msec, and no cardiac adverse events with PYR + PQP or either drug plus placebo [18].

Given the long half‐lives of both drugs, the potential for cumulative QTc prolongation with monthly PYR + PQP dosing is a key concern for risk:benefit evaluation. Conducting long‐term studies in healthy volunteers is impractical; thus, concentration–QTc modeling was used to simulate an alternative chemoprevention regimen with once‐daily dosing for 2 days monthly over 6 months. Simulations indicated a slight increase in ΔΔQTcF after the final versus initial dose, but the 90% CIs overlapped, suggesting no cumulative QTcF risk. This is consistent with clinical data, which showed no cumulative cardiotoxicity with monthly DHA‐PQP over 3 months in healthy individuals aged 3–60 years [26].

Although PQP exposure increased with PYR coadministration, no mechanistic basis for this interaction is established. PYR does not inhibit known PQP‐metabolizing enzymes in vitro, and clinical data do not suggest an interaction. Although PQP inhibits one metabolic pathway of PYR, physiologically based PK modeling predicted no significant impact (N. Abla, personal communication). PQP shows variable PK and interactions with other antimalarials, including ganaplacide, artefenomel, and cipargamin [14, 27, 28]. Combining PQP with ganaplacide raised PQP *C*_max_ by ~70% without changing AUC and slightly increased ΔQTcF [27]. With artefenomel, PQP PK remained stable, but artefenomel exposure rose up to 1.7‐fold, and ΔΔQTcF increased to 22.1 msec versus 17.9 msec for PQP alone [14].

The final model suggested a flattening of the concentration–ΔQTcF relationship at higher PQP exposures. This is in contrast to previous reports in healthy adults, where a linear relationship was described, based on simple linear regression [29], linear fixed‐effect models with treatment as the fixed effect [27, 28], or a linear mixed‐effect model [14]. Notably, previous studies did not meet ICH E14 requirements for high‐resolution ECGs, dense PK sampling, and covariate control. ICH E14‐compliant models must also consider more robust modeling strategies which can explore *E*_max_ relationships and detect a plateau effect at high concentrations. This Phase 1 PYR + PQP study was specifically designed to ensure rich data were available to support concentration–QTc modeling conforming to ICH E14 recommendations [18]. The design was efficient, with a sample size of just 37 patients providing paired time‐matched PK and digitalized triplicate ECG data. Thus, previous evaluations may have had limited data at higher concentrations and/or used methods that were unable to detect non‐linear effects. There is some evidence from population PK analysis in African malaria patients that an *E*_max_ function better describes the PQP–QTc relationship than a linear model, likely due to the large sample size and broad concentration range [30]. However, our findings should not be interpreted as evidence of a saturable QTc effect across all clinical contexts, and caution is warranted when extrapolating beyond the current data.

Few concentration–QTc modeling studies have been conducted for malaria treatments [14, 31, 32]. To our knowledge, the current study was unique in that both PYR and PQP effects were considered separately and in combination. Previous studies considered monotherapy [31, 32], or the effect of the drug combination on one partner drug [14]. In this study, both drugs were components of approved antimalarial drugs, allowing evaluation of therapeutic doses; whereas for investigational drugs, supratherapeutic doses may be evaluated, constrained by safety considerations. Designing Phase I studies to generate data for concentration–QTc modeling provides early insights into cardiac risk and is accepted by regulatory agencies for comprehensive risk:benefit evaluation.

To achieve equivalency with the outputs of thorough QT/QTc studies, ICH E14 guidelines specify conducting concentration–QTc modeling in healthy participants using rich data sets [19, 23]. This approach differs from population PK modeling of patient field trials, which, while informative on clinical risk, are limited by disease‐related confounders, sparse sampling, and non‐synchronized ECG and PK data. These limitations can lead to underestimation of QTc effects, especially around individual *C*_max_, highlighting the need for caution when interpreting such analyses.

A limitation of the study was the parallel design, chosen for practicality given the drugs' long half‐lives, though a crossover design would have been preferable. Nevertheless, even considering the parallel design and small sample size, the use of the concentration–QTc approach permitted evaluation of the effect of PYR and PQP on QTcF individually and in combination.

Real‐world variability in PQP PK due to solubility differences between formulations [33] and variable food intake [12], has direct implications for QTc risk and efficacy. PQP PK is highly sensitive to dietary fat. A high‐fat/low‐calorie meal doubled PQP *C*_max_ and increased ΔΔQTcF to 35.9 msec, while a high‐fat/high‐calorie meal tripled *C*_max_ and increased ΔΔQTcF to 46.0 msec [12]. Although maintaining fasting is challenging in malaria‐endemic settings, typical local meals are generally low in fat and may mitigate risk [34]. Notably, in 701 patients infected with malaria and administered DHA‐PQP, there were no reports of proarrhythmia or related signs and symptoms, such as fainting/syncope, palpitations, convulsions/seizures, or chest pain [13]. However, data on local diet effects on PQP exposure and QTc prolongation remain limited. In terms of chemoprevention, monthly dosing of children with DHP‐PQP did not indicate any increase in the incidence or severity of QTc prolongation [35], and though studies in pregnant women are limited, risk appears to decrease with repeated monthly dosing [36].

This study successfully applied a concentration–QTcF modeling approach aligned with ICH E14 guidance to evaluate QT risk from PYR + PQP in healthy volunteers. The model showed no additional QT prolongation with the combination versus PQP alone, despite increased drug exposures. Simulations of monthly dosing suggest no cumulative QTcF risk. This study provides a valuable template for evaluating QT interval prolongation risk early in the clinical development of new antimalarial combination drugs.

## Author Contributions

All authors wrote the manuscript. M.F., S.C., and I.B.‐F. designed the research. M.F. and N.A. performed the research. M.F., S.C., and I.B.‐F. analyzed the data.

## Conflicts of Interest

I.B.‐F., N.A., and S.C. are employees of MMV Medicines for Malaria Venture. M.F. is an employee of PhinC Development, which received funding from MMV Medicines for Malaria Venture.

## Supporting information


Data S1.

## Data Availability

De‐identified participant data are available on reasonable request and with completion of a signed data access agreement from (https://www.mmv.org/about‐us/contact‐us) referencing this publication. Data will be available for at least 5 years from the publication of this study.
