# Supplementary material for: Concentration–Response Analysis of the Combination of Pyronaridine and Piperaquine on Corrected QT Interval From a Randomized, Double‐Blind, Placebo‐Controlled Study in Healthy Adults of African Sub‐Saharan Origin
Source: Clin Transl Sci. 2025 Jul 21;18(7):e70305. doi: 10.1111/cts.70305 (PMC12278333; doi:10.1111/cts.70305)
Supplement: Supplementary file 1 — Data S1. [file CTS-18-e70305-s001.docx]

**SUPPORTING INFORMATION**

**Concentration–response analysis of the combination of pyronaridine and piperaquine on corrected QT interval from a randomized, double-blind, placebo-controlled study in healthy adults of African sub-Saharan origin**

*Mathieu Felices, Isabelle-Borghini-Fuhrer, Nada Abla, Stephan Chalon^*^*

**Contents**

Equation S1. Mixed linear model used as a starting point and alternatives 3

Figure S1. Drug effect on heart rate (HR) evaluated by time course of (A) HR; (B) change in HR from baseline (ΔHR); and (C) placebo corrected change in HR from baseline (ΔΔHR) 4

Figure S2. Adequacy of QT correction evaluated graphically by QTcF–HR quantile plots with lineal mixed effects line (95%CI) 5

Figure S3. Investigation of hysteresis for placebo-corrected QTcF change from baseline (ΔΔQTcF) and (A) pyronaridine concentrations; and (B) piperaquine concentrations 6

Figure S4. Evaluation of drug effect by time course of (A) QTcF; (B) the change in QTcF versus baseline (ΔQTcF); and (C) placebo-corrected ΔQTcF (ΔΔQTcF) 7

Figure S5. Evaluation of the linearity and heterogeneity assumptions between QTc and drug concentrations by scatter plots of the change in QTcF from baseline (ΔQTcF) for (A) pyronaridine concentrations; and (B) piperaquine concentrations 8

Figure S6. Mean estimated and observed QTcF change from baseline (ΔQTcF) with 90% confidence intervals by deciles of concentration for the linear model for pyronaridine and piperaquine illustrating model inappropriateness 9

Table S1. Final model parameter estimates. 11

Figure S7. Diagnostic plots for the final concentration–QTcF model for predictions versus observed values for (A) conditional predictions and (B) non-conditional predictions. 12

Methods S1. Method for prediction of ΔΔQTcF at maximal exposure by treatment arm 13

Equation S1. Mixed linear model used as a starting point and alternatives

$\Delta{QTcF}_{ijkl}=\left( \theta_{0}+\eta_{0,i} \right)+\left( \theta_{1}+\eta_{1,i} \right){PQP}_{ijkl}+{(\theta}_{2}+\eta_{2,i}){PYR}_{ijkl}+\theta_{3,j}{TRT}_{j}+\theta_{4,k}{Day}_{k}+\theta_{5,l}Time+\theta_{6}\left( {QTcF}_{ijk=1,l=0}-\bar{{QTcF}_{0}} \right)+\varepsilon_{ijkl}$

Where:

- $i$ is the subject, $j$ the treatment, $k$ the day and $l$ the time;
- $\Delta{QTcF}_{ijkl}$ is the change from baseline in QTcF for subject $i$ in treatment $j$ at day $k$ and time $l$;
- $\theta_{0}$ is the population mean intercept in the absence of a treatment effect;
- $\eta_{0i}$is the between-subject random effect associated with the intercept term $\theta_{0}$ with mean 0 and variance ${\omega_{0}}^{2}$; and $\eta_{1,i}$is the between-subject random effect associated with the intercept term $\theta_{1}$ with mean 0 and variance ${\omega_{1}}^{2}$; and $\eta_{2i}$is the between-subject random effect associated with the intercept term $\theta_{2}$ with mean 0 and variance${\omega_{2}}^{2}$;
- $\theta_{1}$ is the population mean slope of the assumed linear association between PQP concentration and $\Delta{QTcF}_{ijkl}$ ; and $\theta_{2}$ is the population mean slope of the assumed linear association between PYR concentration and $\Delta{QTcF}_{ijkl}$;
- ${PQP}_{ijkl}$ is the PQP concentration for subject $i$ in treatment $j$ at day $k$ and time $l$; and ${PYR}_{ijkl}$ is the PQP concentration for subject $i$ in treatment $j$ at day $k$ and time $l$;
- ${TRT}_{j}$ is the factor with $j$=4 levels associated to treatment (placebo+placebo, PYR+placebo, PQP+placebo and PYR+PQP);
- $\theta_{3,j}$ is a vector of $j$−1 ($j$=4) parameters associated to treatment factor ${TRT}_{j}$;
- $\theta_{4,k}$ is a vector of $k$−1 ($k$=3) parameters associated to the day factor day $k$ with $k$ levels; and
- $\theta_{5,l}$ is a vector of $l$−1 ($l$=number of distinct time points) parameters associated to the time factor time $l$ with $l$ levels, with time defined as the scheduled time after the last dose. A day by time interaction effect was included in the model and retained if statistically significant;
- $\theta_{6}$ is the fixed effect parameter associated with baseline and $\left( \bar{{QTcF}_{0}} \right)$ is the mean of all baseline;
- $\varepsilon_{ijkl}$ is the residual error with mean 0 and variance $\sigma^{2}$.

If the linear relationship between ΔQTcF and any analyte could not be accepted, an alternative model for the treatment component was considered, replacing the linear contribution with an E_max_ contribution:

$$\frac{(\theta_{j,1}+\eta_{j, 1,i}){Conc}_{ijk}^{\gamma}}{(\theta_{j,2}+\eta_{j,2,i})^{\gamma}+{Conc}_{ijk}^{\gamma}}$$

Where the *j* is the analyte (PQP, PYR), *i* is the subject, $\theta_{j,1}$ is the maximum asymptotic effect due to treatment (E_max_), $\theta_{j,2}$ is the concentration at which the effect is half $\theta_{j,1}$ (EC_50_) and $\eta$ terms are between-subject random effects.

Figure S1. Drug effect on heart rate (HR) evaluated by time course of (A) HR; (B) change in HR from baseline (ΔHR); and (C) placebo corrected change in HR from baseline (ΔΔHR)

Treatments: pyronaridine (PYR)+placebo; piperaquine (PQP)+placebo; PYR+PQP; placebo+placebo, once daily for three days.


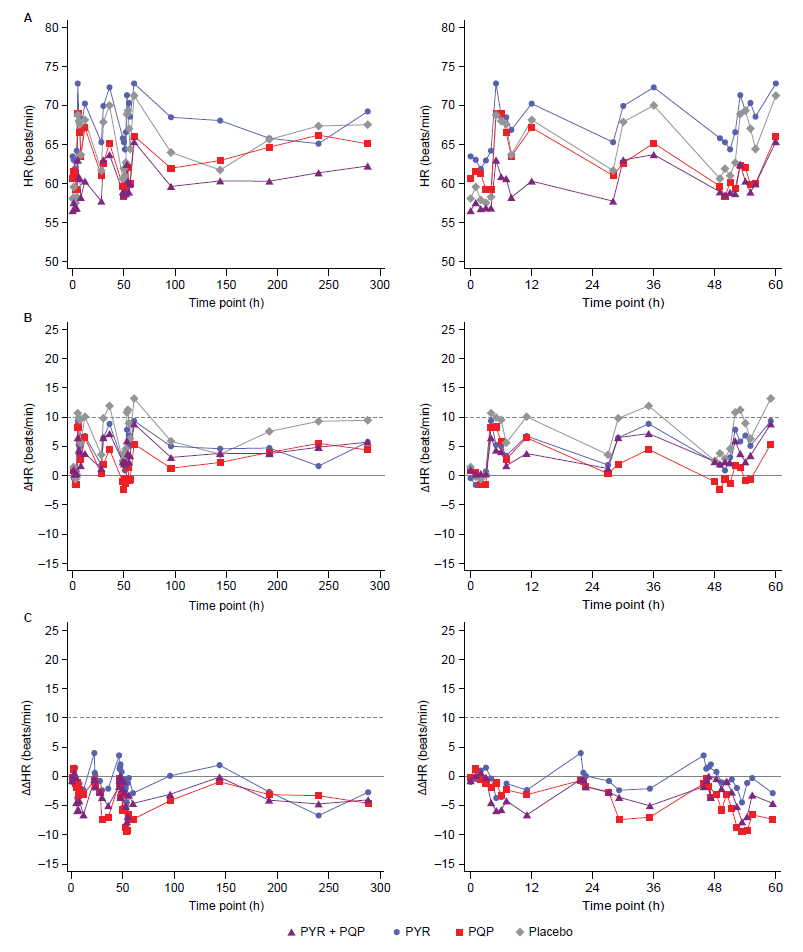


Figure S2. Adequacy of QT correction evaluated graphically by QTcF–HR quantile plots with lineal mixed effects line (95%CI)

Treatments: pyronaridine (PYR)+placebo; piperaquine (PQP)+placebo; PYR+PQP; placebo+placebo, once daily for three days.


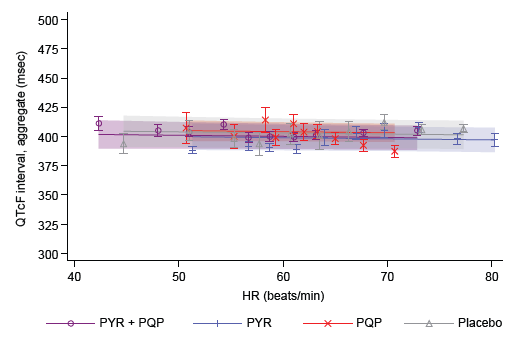


Figure S3. Investigation of hysteresis for placebo-corrected QTcF change from baseline (ΔΔQTcF) and (A) pyronaridine concentrations; and (B) piperaquine concentrations

Treatments: pyronaridine (PYR)+placebo; piperaquine (PQP)+placebo; PYR+PQP; placebo+placebo, once daily for three days.


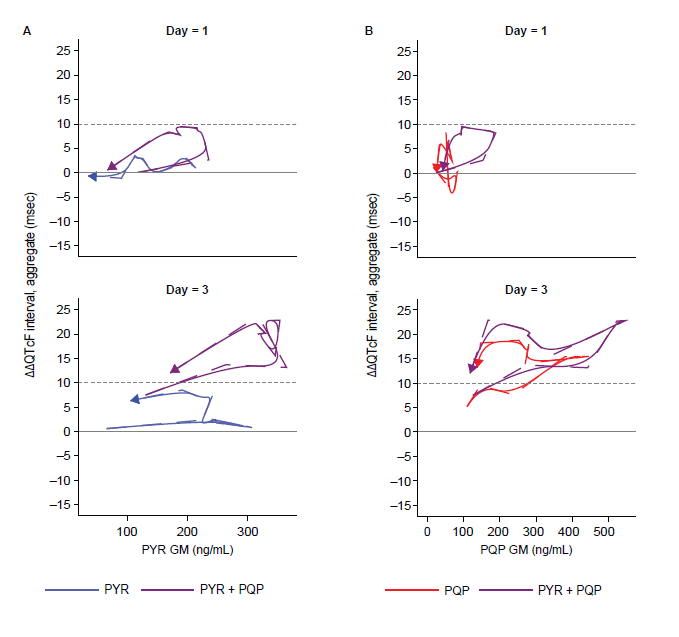


| exposure-normalized Glomb-Ring Index (eGRI) | eGRI |
| --- | --- |
| PYR Day 1: 0.06, PYR Day 3:−1.05  PYR+PQP Day 1: 2.42, PYR+PQP Day 3: −9.26 | PQP Day 1: 0.54, PQP Day 3: −0.08  PYR+PQP Day 1: 3.85, PYR+PQP Day 3: 0.87 |

Figure S4. Evaluation of drug effect by time course of (A) QTcF; (B) the change in QTcF versus baseline (ΔQTcF); and (C) placebo-corrected ΔQTcF (ΔΔQTcF)

Treatments: pyronaridine (PYR)+placebo; piperaquine (PQP)+placebo; PYR+PQP; placebo+placebo, once daily for three days.


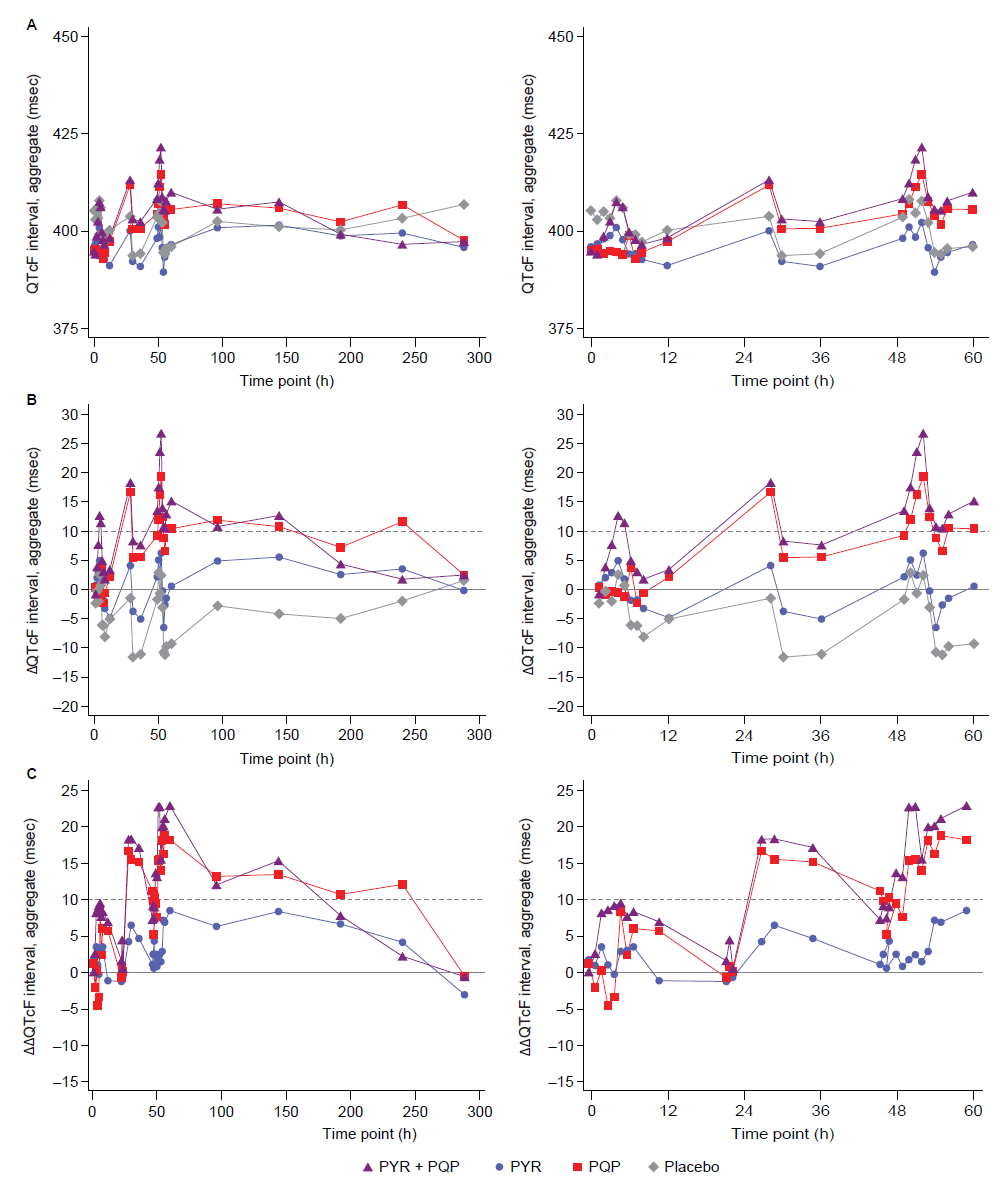


Figure S5. Evaluation of the linearity and heterogeneity assumptions between QTc and drug concentrations by scatter plots of the change in QTcF from baseline (ΔQTcF) for (A) pyronaridine concentrations; and (B) piperaquine concentrations

Treatments: pyronaridine (PYR)+placebo; piperaquine (PQP)+placebo; PYR+PQP; placebo+placebo, once daily for three days. NB: Divergence between the LOESS curve and a linear curve in a concentration–QTc plot, indicates that the true relationship between concentration and QTc change is not linear.


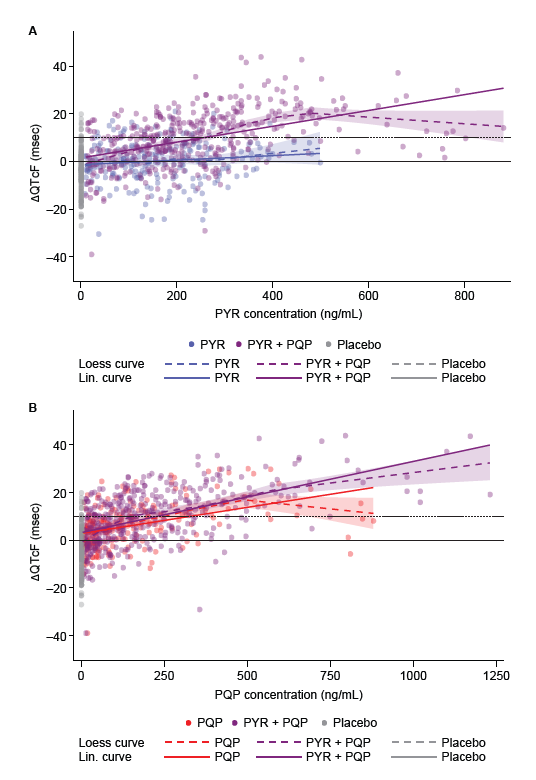


Figure S6. Mean estimated and observed QTcF change from baseline (ΔQTcF) with 90% confidence intervals by deciles of concentration for the linear model for pyronaridine and piperaquine illustrating model inappropriateness

Treatments: pyronaridine (PYR)+placebo; piperaquine (PQP)+placebo; PYR+PQP; placebo+placebo, once daily for three days.


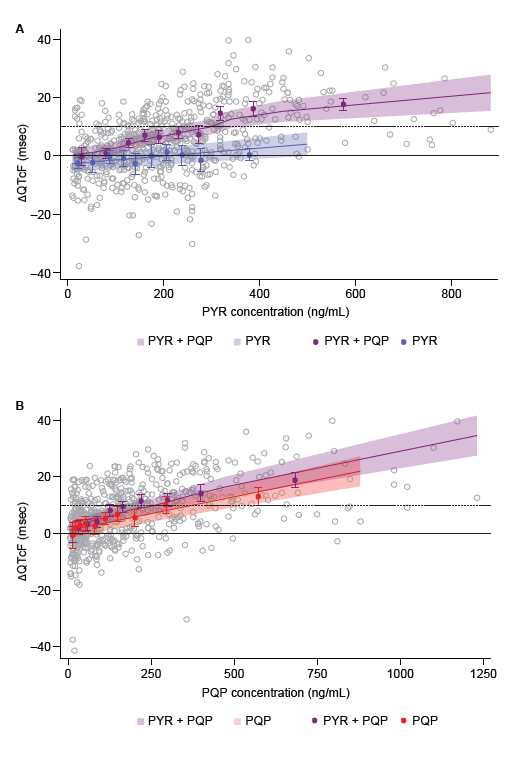


**Equation S2. Final model equation.**

$${\Delta QTcF}_{\boldsymbol{ijkl}}\boldsymbol{=}\left( \theta_{0}+\eta_{0,i} \right)+\frac{\left( \theta_{1}^{1}+\eta_{1,i} \right){PQP}_{ijkl}}{(\theta_{1}^{2}+{PQP}_{ijkl})}+\left( \theta_{2}+\eta_{2,i} \right){PYR}_{ijkl}+\theta_{3,j}{TRT}_{j}+\theta_{4,k,l}{Day}_{k}\times{Time}_{l}+\theta_{6}({QTcF}_{ijk=1,l=0}-\bar{{QTcF}_{0}})+\varepsilon_{ijkl}$$

Where:

- $i$ is the subject, $j$ the treatment, $k$ the day and $l$ the time;
- $\Delta{QTcF}_{ijkl}$ is the change from baseline in QTcF for subject $i$ in treatment $j$ at day $k$ and time $l$;
- $\theta_{0}$ is the population mean intercept in the absence of a treatment effect;
- $\eta_{0i}$is the between-subject random effect associated with the intercept term $\theta_{0}$ with mean 0 and variance ${\omega_{0}}^{2}$; and $\eta_{1,i}$is the between-subject random effect associated with the intercept term $\theta_{1}$ with mean 0 and variance ${\omega_{1}}^{2}$; and $\eta_{2i}$is the between-subject random effect associated with the intercept term $\theta_{2}$ with mean 0 and variance${\omega_{2}}^{2}$;
- $\theta_{1}^{1}$ and $\theta_{1}^{2}$ are respectively the E_max_ and EC_50_ parameters for the PQP contribution;
- $\theta_{2}$ is the population mean slope of the assumed linear association between PYR concentration and $\Delta{QTcF}_{ijkl}$;
- ${PQP}_{ijkl}$ is the PQP concentration for subject $i$ in treatment $j$ at day $k$ and time $l$; and ${PYR}_{ijkl}$ is the PYR concentration for subject $i$ in treatment $j$ at day $k$ and time $l$;
- ${TRT}_{j}$ is the factor with $j$=4 levels associated to treatment (placebo+placebo, PYR+placebo, PQP+placebo and PYR+PQP);
- $\theta_{3,j}$ is a vector of $j$−1 ($j$=4) parameters associated to treatment factor ${TRT}_{j}$;
- $\theta_{4,k,l}$ are the parameters accounting for the day-by-time interaction for day $k$ with $k$=3 levels and time $l$ with $l$ levels, with time defined as the scheduled time after the last dose;
- $\theta_{6}$ is the fixed-effect parameter associated with baseline and $\left( \bar{{QTcF}_{0}} \right)$ is the mean of all baseline;
- $\varepsilon_{ijkl}$ is the residual error with mean 0 and variance $\sigma^{2}$.

Between-subject random effects were supported on intercept ($\theta_{0}$), PYR slope ($\theta_{1}$) and PQP E_max_ ($\theta_{1}^{1}$ ), with covariances estimated only between the random effect for E_max_ and other random effects, but not between the intercept and the PYR slope random effects which were statistically not different from 0 but retained in the model.

Table S1. Final model parameter estimates.

| Parameter | Estimate | SE | DF | t value | *P* > \|t\| | 95%CI | | Gradient |
| --- | --- | --- | --- | --- | --- | --- | --- | --- |
| $\theta_{0}$ Intercept (placebo, Day 3, Time 456 h), msec | –3.2369 | 2.2551 | 34 | –1.44 | 0.1603 | –7.8199 | 1.3460 | 0.89964 |
| $\theta_{6}$ Baseline covariate (msec) | –0.1161 | 0.04551 | 34 | –2.55 | 0.0154 | –0.2086 | –0.02363 | –0.02310 |
| $\theta_{2}$ PYR slope (msec per ng/mL) | 0.009375 | 0.004104 | 34 | 2.28 | 0.0287 | 0.001035 | 0.01771 | 0.20794 |
| $\theta_{1}^{1}$ PQP Emax (msec) | 25.6571 | 4.0974 | 34 | 6.26 | <.0001 | 17.3303 | 33.9840 | 0.32364 |
| $\theta_{2}^{1}$ PQP EC50 (ng/mL) | 300.00 | 118.97 | 34 | 2.52 | 0.0165 | 58.2206 | 541.78 | –0.00515 |
| $\theta_{3,PYR}$ Treatment specific intercept PYR (msec) | 1.9266 | 2.6135 | 34 | 0.74 | 0.4661 | –3.3848 | 7.2380 | 0.057480 |
| $\theta_{3,PQP}$ Treatment specific intercept PQP (msec) | 3.4972 | 2.7241 | 34 | 1.28 | 0.2079 | –2.0389 | 9.0333 | 0.43071 |
| $\theta_{3,PYR+PQP}$ Treatment specific intercept PYR+PQP (msec) | 2.5538 | 2.5886 | 34 | 0.99 | 0.3308 | –2.7069 | 7.8145 | 0.68122 |
| **Day (D)–by–time (T)** |  |  |  |  |  |  |  |  |
| TH_D1_T1 | –1.2460 | 1.5567 | 34 | –0.80 | 0.4290 | –4.4095 | 1.9175 | 0.090108 |
| TH_D1_T2 | –2.7788 | 1.6436 | 34 | –1.69 | 0.1000 | –6.1191 | 0.5614 | 0.049326 |
| TH_D1_T3 | –1.7614 | 1.6579 | 34 | –1.06 | 0.2955 | –5.1305 | 1.6078 | 0.030690 |
| TH_D1_T4 | 1.7022 | 1.6364 | 34 | 1.04 | 0.3056 | –1.6233 | 5.0277 | 0.032304 |
| TH_D1_T5 | 1.1096 | 1.6204 | 34 | 0.68 | 0.4981 | –2.1835 | 4.4026 | 0.057202 |
| TH_D1_T6 | –1.6466 | 1.5983 | 34 | –1.03 | 0.3102 | –4.8947 | 1.6014 | 0.058174 |
| TH_D1_T7 | –3.7704 | 1.6086 | 34 | –2.34 | 0.0251 | –7.0394 | –0.5014 | 0.059851 |
| TH_D1_T8 | –4.1174 | 1.5925 | 34 | –2.59 | 0.0142 | –7.3537 | –0.8811 | 0.061333 |
| TH_D1_T12 | –1.7441 | 1.5624 | 34 | –1.12 | 0.2721 | –4.9193 | 1.4312 | 0.076453 |
| TH_D1_T24 | 3.4332 | 1.5319 | 34 | 2.24 | 0.0317 | 0.3200 | 6.5465 | 0.079680 |
| TH_D2_T4 | 3.4017 | 1.7111 | 34 | 1.99 | 0.0549 | –0.07576 | 6.8791 | 0.010601 |
| TH_D2_T6 | –3.7884 | 1.7080 | 34 | –2.22 | 0.0333 | –7.2594 | –0.3174 | 0.051470 |
| TH_D2_T12 | –2.1595 | 1.6645 | 34 | –1.30 | 0.2032 | –5.5422 | 1.2232 | 0.065941 |
| TH_D2_T24 | 6.5480 | 1.6351 | 34 | 4.00 | 0.0003 | 3.2251 | 9.8709 | 0.041435 |
| TH_D3_T1 | 0.7085 | 1.7043 | 34 | 0.42 | 0.6802 | –2.7551 | 4.1722 | 0.011746 |
| TH_D3_T2 | 1.8992 | 1.7525 | 34 | 1.08 | 0.2861 | –1.6624 | 5.4608 | –0.00092 |
| TH_D3_T3 | 3.3461 | 1.7469 | 34 | 1.92 | 0.0639 | –0.2041 | 6.8962 | –0.02146 |
| TH_D3_T4 | 6.2049 | 1.7522 | 34 | 3.54 | 0.0012 | 2.6439 | 9.7659 | –0.02122 |
| TH_D3_T5 | –0.8241 | 1.7300 | 34 | –0.48 | 0.6368 | –4.3398 | 2.6915 | 0.009269 |
| TH_D3_T6 | –4.7889 | 1.7200 | 34 | –2.78 | 0.0087 | –8.2844 | –1.2934 | 0.011157 |
| TH_D3_T7 | –4.8224 | 1.7343 | 34 | –2.78 | 0.0088 | –8.3468 | –1.2979 | 0.009436 |
| TH_D3_T8 | –2.1161 | 1.7221 | 34 | –1.23 | 0.2276 | –5.6158 | 1.3836 | 0.013541 |
| TH_D3_T12 | 1.2457 | 1.7120 | 34 | 0.73 | 0.4718 | –2.2335 | 4.7248 | 0.046449 |
| TH_D3_T24 | 3.4021 | 1.6426 | 34 | 2.07 | 0.0460 | 0.06406 | 6.7402 | 0.042570 |
| TH_D3_T72 | 2.0427 | 1.5565 | 34 | 1.31 | 0.1982 | –1.1205 | 5.2058 | 0.066565 |
| TH_D3_T120 | 1.6257 | 1.5210 | 34 | 1.07 | 0.2927 | –1.4652 | 4.7167 | 0.092444 |
| TH_D3_T288 | –1.2589 | 1.5212 | 34 | –0.83 | 0.4137 | –4.3503 | 1.8326 | 0.081616 |
| **Variance components** |  |  |  |  |  |  |  |  |
| Residual | 42.1360 | 2.0920 | 34 | 20.14 | <.0001 | 37.8844 | 46.3875 | 0.65673 |
| Between subject variance (intercept) | 19.2559 | 5.5471 | 34 | 3.47 | 0.0014 | 7.9828 | 30.5290 | –0.06999 |
| Between subject variance (slope PYR) | 0.000146 | 0.000111 | 34 | 1.31 | 0.1993 | –0.00008 | 0.000372 | –49.8188 |
| Covariance (intercept, E_max_ PQP) | 0.1169 | 6.7400 | 34 | 0.02 | 0.9863 | –13.5804 | 13.8142 | –0.09163 |
| Covariance (slope PYR, E_max_ PQP) | –0.02719 | 0.03204 | 34 | –0.85 | 0.4020 | –0.09230 | 0.03792 | 0.13668 |
| Between subject variance (E_max_ PQP) | 10.4294 | 12.9410 | 34 | 0.81 | 0.4259 | –15.8700 | 36.7287 | –0.14039 |

CI, confidence interval; DF, degrees of freedom; SE, standard error.

Figure S7. Diagnostic plots for the final concentration–QTcF model for predictions versus observed values for (A) conditional predictions and (B) non-conditional predictions.

Treatments: pyronaridine (PYR)+placebo; piperaquine (PQP)+placebo; PYR+PQP; placebo+placebo, once daily for three days.


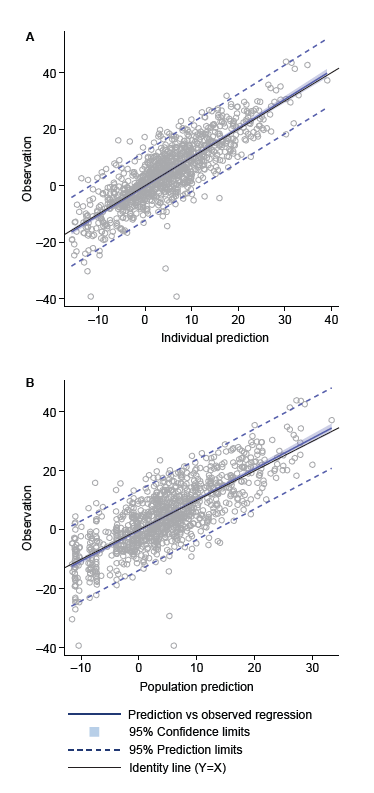


Methods S1. Method for prediction of ΔΔQTcF at maximal exposure by treatment arm

The final model parameters were used to compute predicted ΔΔQTcF (SE) and 90%CIs at the GM *C*_max_ on day 1 and day 3 as follows:

PYR-placebo treatment group:

$$\theta_{3,PYR}+\theta_{2}\times\mathrm{GM}(C_{max, PYR})$$

PQP-placebo treatment group:

$$\theta_{3PQP}+\theta_{1}^{1}\times GM(C_{maxPQP})/(\theta_{1}^{2}+GM(C_{max,PQP})$$

PYR+PQP treatment group:

- For PYR:

$$\theta_{3,PYR+PQP}+\theta_{2}\times GM(C_{max,PYR})+\theta_{1}^{1}\times Mean(PQP conc. at PYR T_{max})/(\theta_{1}^{2}+\mathrm{Mean}\left( PQP conc at PYR T_{max} \right))$$

- For PQP:

$$\theta_{3,PYR+PQP}+\theta_{2}\times\mathrm{Mean}\left( PYP conc. at PQP T_{max} \right)+\theta_{1}^{1}\times GM(C_{max,PQP})/(\theta_{1}^{2}+GM(C_{max,PQP}))$$

Due to the non-linearity of the model, the SE for the predictions used to compute the 90%CI were determined using the delta method.
